# Hemophilic Arthropathy of the Knee and Its Association with Reduced Muscle Strength and Activation and the Pressure Pain Threshold: A Case-Control Study

**DOI:** 10.3390/jcm12093275

**Published:** 2023-05-04

**Authors:** Mar Villalón-González, Íñigo Fernández de Luco-Santamaría, Rubén Cuesta-Barriuso, José Antonio López-Pina, Raúl Pérez-Llanes

**Affiliations:** 1 Department of Physiotherapy, University of Murcia, 30100 Murcia, Spain; mariamar.villalong@um.es; 2 Department of Physiotherapy, Catholic University San Antonio-UCAM, 30107 Murcia, Spain; ifernandez2@ucam.edu (Í.F.d.L.-S.); rperez@ucam.edu (R.P.-L.); 3 Department of Surgery and Medical-Surgical Specialties, University of Oviedo, 33006 Oviedo, Spain; 4 Department of Basic Phycology and Methodology, University of Murcia, 30100 Murcia, Spain; jlpina@um.es

**Keywords:** hemophilia, knee arthropathy, quadriceps strength, electromyography, pressure pain threshold

## Abstract

(1) Background: Hemophilia is characterized by recurrent hemarthrosis leading to degenerative arthropathy. The aim was to evaluate the differences in muscle strength and activity and the pressure pain threshold between patients with knee arthropathy and their healthy peers; (2) Methods: A case-control study in which 23 adult patients with knee arthropathy and 24 healthy peers matched in terms of characteristics were recruited. The study variables were quadriceps muscle strength, muscle activation and the pressure pain threshold; (3) Results: There were significant differences between the two groups in quadriceps strength on the dominant (CI95%: 64.69, 129.2) and non-dominant (CI95%: 29.95, 93.55) sides and in the pressure pain threshold on the dominant (CI95%: 3.30, 43.54) and non-dominant (CI95%: 3.09, 45.25) sides. There were differences in neuromuscular fatigue on the non-dominant side in the vastus medialis (CI95%: 8.72, 21.51), vastus lateralis (CI95%: 4.84, 21.66) and rectus femoris (CI95%: 6.48, 24.95) muscles; (4) Conclusions: Muscle strength and the pressure pain threshold are lower in patients with hemophilia. Quadriceps muscle activation in patients with hemophilic knee arthropathy does not in any way differ from activation in healthy subjects. However, muscle fatigue is greater in patients with knee arthropathy. Strength training in patients with hemophilia should focus on the activation of the vastus medialis and lateralis muscles.

## 1. Introduction

Hemophilia is a hematological pathology characterized by musculoskeletal signs and symptoms. Hemarthrosis is the most relevant clinical manifestation, most prevalent in knees, elbows and ankles [1]. The recurrence of hemarthrosis causes progressive and degenerative hemophilic arthropathy. This arthropathy is characterized by chronic pain, periarticular muscle atrophy, a limited range of motion and axial disorders. The onset and establishment of this degenerative joint injury causes medium- and long-term disability, affecting the patient’s perceived quality of life [2].

Although the prophylactic administration of the missing clotting factor is the gold standard in the prevention of hemarthrosis and hemophilic arthropathy [3], adult patients or those without access to prophylaxis present joint sequelae from an early age [4,5]. The main complication posed by drug treatment is the development of antibodies to the clotting factors (inhibitors) [5]. These inhibitors increase the frequency of hemarthrosis and lead to a greater degree of severity in joint sequelae [6].

Muscle strength is the ability to generate tension in a muscle or muscle group to counter or overcome a counterforce [7]. It has been noted [6,8,9] that muscle strength has a predictive ability on the onset of disability. Muscle mass and strength and physical performance are markers that identify a decline in the performance of the activities of daily living [6]. Hemophilic arthropathy is characterized not only by decreased mobility but also by a deterioration of muscle strength in the upper and lower limbs [10].

The pressure pain threshold can adversely affect the generation of strength, preventing maximum muscle benefit from being achieved due to a lower tolerance to pain [11]. In patients with chronic pain, a 20–30% reduction in strength in the painful limb is considered normal [12]. This phenomenon may be due to typical avoidance behavior or kinesiophobia in these patients. This avoidance behavior can, in turn, lead to physiological changes in the limb such as muscle atrophy [12]. Avoidance can also lead to qualitative changes in muscle contraction, such as abnormal coordination, resulting in ineffective contractions that reduce muscle strength [12]. This disability contributes to the vicious circle in the deterioration of joint function [13].

To date, no study has compared muscle strength and activation in patients with hemophilia against that of their healthy pairs. Our study hypothesis is that muscle strength, the degree of muscle activation and muscle fatigue are greater in healthy subjects than in those with hemophilic arthropathy. The aim of the present study was to establish the differences in muscle strength, muscle activity and the pressure pain threshold between patients with bilateral hemophilic knee arthropathy and their healthy peers, depending on the development of inhibitors.

## 2. Materials and Methods

### 2.1. Design

A cross-sectional descriptive case-control study was carried out following the guidelines for the reporting of observational studies according to Strengthening the Reporting of OBservational Studies in Epidemiology (STROBE) [14].

### 2.2. Ethical Considerations

This study was approved by the Medical Research Ethics Committee of the Virgen de la Arrixaca University Hospital of Murcia (ID code: 2021-9-10-HCUVA). The study conforms to the Declaration of Helsinki. All patients who met the selection criteria signed an informed consent document. The study was prospectively registered in the International Registry of Clinical Trials (ClinicalTrials.gov ID: NCT05589662).

### 2.3. Participants

Patients with hemophilia were recruited from the hemophilia associations of the regions of Murcia and Malaga. The healthy subjects were volunteers from the region of Murcia. The date of sample recruitment was December 2022. Data collection was carried out in January 2023.

The criteria for inclusion of patients with hemophilia were as follows: (i) a diagnosis of severe hemophilia A or B; (ii) over 18 years of age; (iii) a medical diagnosis of bilateral knee arthropathy and a score of more than 4 points on the Hemophilia Joint Health Score [15]; (iv) not having suffered knee hemarthrosis in the 12 months preceding the study; (v) and having an autonomous walking ability, without needing technical aids. Healthy subjects met the following criteria: (i) no knee joint injury at the time of evaluation; (ii) over 18 years of age; (iii) no previous knee injuries in the 12 months prior to evaluation; and (iv) normal physical activity (neither sedentary nor very active in terms of sport). Normal physical activity was established according to the criteria of the World Health Organization (150 min per week of moderate aerobic physical activity, 75 min per week of vigorous aerobic physical activity, or an equivalent combination of moderate and vigorous activities) [16].

The study exclusion criteria were as follows: (i) neurological or cognitive disorders that prevent the understanding of the physical tests; (ii) having undergone orthopedic surgery (total knee replacement, arthroscopic or open synovectomy); (iii) and failure to sign the informed consent document.

### 2.4. Sample Size

The sample size was calculated with the statistical package G*Power (version 3.1.9.2; Heinrich-Heine-Universität Düsseldorf, Germany) based on a previous prospective cohort study [17] (cases) and reliability pilot study (controls). A one-tailed hypothesis, an effect size of 0.80, an error probability of 0.05, a power (1-error probability) of 0.80 and an allocation ratio (N2/N1) of 1 were used for the sample size calculation. A total sample size of 42 subjects was estimated.

### 2.5. Procedures

Patients with hemophilia were recruited from two patient associations from southeast Spain. All patients were asked to complete a pre-screening form with clinical and anthropometric data. Patients with other congenital coagulopathies or without a diagnosis of hemophilic knee arthropathy at the time of the study were not included. The healthy controls were recruited from among the teaching and research staff of the San Antonio Catholic University of Murcia (Spain). Homogeneity between the groups was achieved by age-matched quotas of the patients with hemophilia and the healthy controls.

### 2.6. Outcome Measures

The main anthropometric data (weight, height and body mass index) and age of the participants were collected, as well as clinical data of the group of patients with hemophilia (joint damage, type of hemophilia, inhibitor development and type of drug treatment). Joint condition was measured with the hemophilia-specific scale, the Hemophilia Joint Health Score [15]. It covers eight items: swelling and its duration, pain, atrophy and muscle strength, crepitus, and loss of flexion and extension. It scores from 0 to 20 points (maximum joint damage) per joint. Joint damage in both knees was evaluated (range of 0–40).

The primary variable was quadriceps muscle strength. The secondary variables were the pressure pain threshold and quadriceps muscle activation. All evaluations were performed by a physiotherapist with more than 20 years of clinical experience. The dominant side of the body was determined by asking the patient which leg was preferred for kicking a ball or performing any voluntary leg movement. Each patient became familiarized with each measuring instrument one day before data collection.

Quadriceps muscle strength was measured with a pressure dynamometer (Lafayette Manual Muscle Tester 01165) [18]. This device measures in Newton the force exerted by the patient in the muscle movement prompted. The higher the value, the greater the muscle strength. The participants were placed in a sitting position with 90-degree hip flexion and 75-degree knee flexion. The dynamometer was placed perpendicular to the leg to be evaluated, above the lateral malleolus (maintaining knee flexion). The participants were asked to perform two 5 s isometric maximum contractions against the dynamometer held by the rater. There was a 30 s break between contractions. The average value between the measurements obtained was used [19].

The pressure pain threshold was measured using a pressure algometer (model Wagner FDIX, Wagner Instruments, Greenwich, CT, USA). This device measures in Newton/cm^2^ the pressure points at which the subject perceives pain. Pressure was applied to the chosen point and was progressively increased at an approximate speed of 50 kPa/s, until the patient reported that the sensation began to be painful [20]. The pain threshold was measured bilaterally, 3 cm medially to the midpoint of the inner edge of the patella and 2 cm proximally from the upper pole of the patella [20]. To avoid tissue damage, the evaluations were carried out at 5 min intervals between measurements.

Muscle electrical activity and its activation level was measured with surface electromyography (surface EMG; Shimmer Sensing, Dublin, Ireland) [21]. The electrodes used in the evaluation (model Ambu^®^ WhiteSensor 4200) were silver/silver chloride (Ag/AgCl) bipolar. These electrodes, rectangular in shape, measured 28 × 44 mm, with a measuring area of 46 mm^2^, located at a distance of 2 cm [22]. The electrodes were placed longitudinally to the muscle fibers, with a reference electrode at a distance (anterior tibial tuberosity). In order to achieve maximum muscle strength, the rater used the same verbal command to prompt each contraction. The subject’s skin was prepared according to the recommendations of the SENIAM project (Surface EMG for a Non-Invasive Assessment of Muscles) [23]. Depending on the muscle studied, the electrodes were placed in different locations in (i) the vastus medialis (80% of the line between the anterior superior iliac spine (ASIS) and knee interline); (ii) the vastus lateralis (2/3 of the line between the ASIS and lateral patella facet); (iii) and the rectus femoris (50% of the line between the ASIS and superior pole of the patella). The unit of measurement is µV. The higher the score, the greater the muscle activation.

The electromyographic evaluation protocol described by Skou et al. was used [20]. Due to the restricted range of motion in some patients, all subjects were evaluated in the same way: a supine position with 75-degree hip and knee flexion.

### 2.7. sEMG Analysis

The reliable and validated surface electromyography (sEMG) mDurance^®^ system (mDurance Solutions SL, Granada, Spain) was used to record muscle activity during a functional task (ICC = 0.91; 95%CI = 0.83–0.95) [24].

The mDurance^®^ system consists of three parts: (a) a Shimmer3 EMG unit (Realtime Technologies LtD, Dublin, Ireland), which is a bipolar surface electromyography sensor for the acquisition of muscle activity. Each Shimmer3 has two channels, with a sampling rate of 1024 Hz. Shimmer3 applies an 8.4 Hz bandwidth, with an EMG signal resolution of 24 bits and an overall amplification of 100–10,000 *v*/*v*; (b) The mDurance Android application, which receives data from the Shimmer3 and sends it to a cloud service; (c) The mDurance cloud service where data are stored, filtered and analyzed [24].

For the processing and filtering of the raw data, a fourth-order Butterworth bandpass filter with a cut-off frequency of 20–450 Hz was used. The signal was smoothed using a window size of 0.025 s root mean square (RMS) and an overlapping of 0.0125 s between windows [24]. The variables extracted from this portion of the signal were the mean RMS and median frequency. The values of all repetitions from the same subject and test were averaged. The mean RMS was expressed in the microV (µV) of the middle third of the isometric contraction. The beginning and end of the signal were identified using a threshold method and verified visually afterwards. The variation median frequency (VMF) is defined as that frequency that divides the power density spectrum into two regions with the same amount of power.

Prior to recruitment, the intraobserver reliability was calculated, measuring the study variables in a sample of six people, who were not included in the study. A high intraobserver reliability was noted in quadriceps muscle strength (ICC = 0.94) and the knee pressure pain threshold (ICC = 0.90). In terms of surface electromyography reliability, we noted a moderate-high reliability in the activation of the vastus medialis (ICC = 0.88) and rectus femoris (ICC = 0.87).

### 2.8. Statistical Analysis

SPSS 19.0 software (IBM SPSS Statistics for Windows; IBM Corp, New York, NY, USA) was used to perform the data analysis. Normality was assessed with the Shapiro–Wilk test. A comparative analysis was made between the two groups. For the parametric data, the mean and standard deviation were calculated, and a Student’s *t*-test was used for independent samples. For the non-parametric data, the median and the interquartile range were calculated, using the Mann–Whitney U test. The effect size of the comparisons between groups was determined by Cohen’s d, which was interpreted as very small (d < 0.20), small (d = 0.20–0.49), medium (d = 0.50–0.79) and large (d > 0.8) effect sizes [25]. The correlation between the knee range of motion, pressure pain threshold and strength variables was obtained using Spearman’s non-parametric test. According to the calculation parameters of the a priori sample size, the statistical significance was set at *p* < 0.05 for a 95% confidence interval (CI).

## 3. Results

Given the possibility that any subject might drop out on the day of the measurements, 47 participants were included in the study. Twenty-three subjects had hemophilia (mean age: 37.39; standard deviation (SD) = 7.04), with an average knee joint damage of 20.43 (SD: 5.19) points. The average age of the 24 healthy peers was 41.50 (SD: 8.15) years. Table 1 shows the descriptive characteristics of the two groups.

When comparing the two groups we found statistically significant differences in quadriceps strength on the dominant (CI95%: 64.69, 129.2; *p* < 0.001; d = 1.38) and non-dominant (CI95%: 29.95, 93.55; *p* < 0.001; d = 1.14) sides. There were also statistically significant differences in the pressure pain thresholds of the two groups on the dominant (CI95%: 3.30, 43.54; *p* = 0.01; d = 0.68) and non-dominant (CI95%: 3.09, 45.25; *p* = 0.03; d = 0.56) sides. The measurement of the variation of median frequency (VMF) disclosed statistically significant differences on the non-dominant side in the vastus medialis (CI95%: 8.72, 21.51; *p* < 0.001; d = 1.18), vastus lateralis (CI95%: 4.84, 21.66; *p* = 0.02; d = 0.68) and rectus femoris (CI95%: 6.48, 24.95; *p* < 0.001; d = 1.01) muscles. On the dominant side, we only observed differences between groups in the fatigue of the rectus femoris muscle (CI95%: 0.76, 14.79; *p* = 0.02; d = 0.64). Table 2 shows the central tendency and dispersion statistics and the differences between groups. Table 3 indicates the effect size of the differences between hemophilia patients and their healthy peers.

In the group of patients with hemophilia, vastus medialis muscle activation was positively correlated (*p* < 0.05) with the activation and variation of median frequency of all the quadriceps muscles. Quadriceps strength was positively correlated with vastus medialis (*p* = 0.02) and vastus lateralis (*p* = 0.01) activation. Knee range of motion was likewise positively correlated with quadriceps strength (*p* = 0.04) but negatively correlated with the variation of median frequency of the vastus medialis muscle (*p* = 0.01). In the same way, there was a positive correlation (*p* < 0.05) between the variation of median frequency values of all the muscles. The controls exhibited a positive correlation (*p* < 0.05) between the activations of all the muscles evaluated. Quadriceps strength was negatively correlated with the variation of median frequency of the vastus medialis muscle (*p* = 0.04). Table 4 shows the correlation analysis between the dependent variables in each of the study groups.

## 4. Discussion

This study assessed the differences in muscle strength, muscle activation and the pressure pain threshold between patients with hemophilic knee arthropathy and their healthy peers, depending on the development of inhibitors. Patients with hemophilia exhibited a lower muscle strength, greater muscle fatigue in the non-dominant limb and a lower pressure pain threshold.

The joint deterioration and chronic pain typically present in hemophilic arthropathy are part of a vicious circle where immobilization produces greater muscle atrophy and vice versa [26]. Immobilization and a sedentary lifestyle, secondary to pain and recurrent hemarthrosis in patients with hemophilia, prevent the improvement of muscle strength. The loss of strength as a result of joint damage and movement limitation is typical in other degenerative diseases such as osteoarthritis [27]. The differences observed in our study regarding quadriceps muscle strength are consistent with the results described by Hilberg et al. [26], who reported decreased isometric strength in the knee extensor mechanism in patients with hemophilia.

Full range of motion strength training produces significantly greater neuromuscular adaptations and hypertrophy in the lower limbs than partial range of motion training [28]. Therefore, the decreased range of motion in the knees, characteristic of hemophilic arthropathy, should be considered one of the main causes of poor muscle strength in these patients. The therapeutic approach to strength in patients with hemophilia should take into account that changes in fasciculus length modify muscle function. The restricted range of motion reduces the number of sarcomeres in series, varying the joint angle of optimal strength. In this way, the strength–length ratio is altered, the shortening velocity is reduced and the strength–velocity ratio is altered [29]. However, a recent review [28] found no great differences in the generation of changes in muscle architecture between full versus partial range of motion training.

There is a very close relationship between muscle activation and the generation of strength. The inability to recruit all the high-threshold motor units produces CNS fatigue. This fatigue translates into a reduction in muscle strength and an alteration in muscle activation during physical activity [30]. Our results disclosed that patients with hemophilic arthropathy exhibited poorer electromyographic activity, compared to healthy subjects. Similarly, we found increased muscle fatigue, especially in the non-dominant limb.

Patients with hemophilic arthropathy maintain their stability in standing position using a greater activation of the knee extensor muscles compared to healthy subjects [31]. This activation could explain why the extensor muscles of hemophilia patients obtain higher fatigue values compared to their healthy peers. The association between a greater alteration of neuromuscular control and increased pain and joint deterioration of the knee extensor mechanism in patients with hemophilia while walking has been described [32]. Muscle fatigue and irregular muscle activation patterns are potential sources of joint stress, increasing the risk of bleeding and also, therefore, degenerative joint damage [33].

The painful experience is influenced by various factors including biological (comorbidities, nociception, inflammation, etc.), psychological (beliefs about pain, previous experiences, expectations, etc.) and social (culture, environment, social support, etc.) factors [34]. Accordingly, establishing a single cause is practically impossible. Despite the remarkable impact of pain on patients with hemophilia, the relevant pathophysiology in hemophilic arthropathy has hardly been studied to date [35]. These patients commonly experience chronic and acute pain simultaneously, which poses a challenge in the evaluation and management of pain [36].

This study reports a decreased pressure pain threshold in patients with hemophilia compared to healthy subjects, both in the dominant and non-dominant limbs. Similar to our study, Hilberg et al. [37] reported a lower pain threshold of the knee and elbow joints in patients with hemophilic arthropathy, compared to healthy controls. Similarly, these authors suggested that the most clinically damaged joints seem to be more sensitive to pain [37]. Other authors [38] found similar results in the ankle joint, correlating pain and joint damage.

Although it has been reported that the pressure pain threshold can affect the generation of strength [11], we only observed a correlation in rectus femoris activation. Such activation during knee extension observed through electromyography is greater than vastus medialis and lateralis activation in isokinetic contractions [39]. Our study revealed that rectus femoris activation in patients with hemophilia was higher than in healthy subjects. This, in contrast to the low activation of the vastus medialis and lateralis, may be caused by the compensatory model of hemophilic arthropathy, characterized by muscle hypotrophy, especially of the vastus medialis. This compensatory system would allow these patients to perform knee extension movements despite periarticular hypotrophy and a reduced range of motion.

For such restricted activation in the vastus medialis and lateralis, interventions should address central pain mechanisms, such as education and descending inhibitory mechanisms [40], such as aerobic and resistance training [13].

The results of the correlation analysis confirm that a decreased range of movement reduces muscle strength, this being associated with the reduced muscle activation. This relationship creates the aforementioned vicious circle, whereby the smaller the range of motion, the lesser the strength and vice versa. As a result of this process, muscle atrophy, a typical condition in patients with hemophilia, develops and eventually leads to joint damage. These results are to be expected, since we have previously described the relationship between ROM and muscle strength and muscle activation. The correlation found in our study is consistent with similar results on muscle strength and activation in the geriatric population [41].

### 4.1. Limitations of the Study

The recruitment of patients in different age brackets, including different groups of patients and controls, would have made it possible to compare the results based on age and different degrees of joint damage. The inclusion of patients with hemophilia and healthy peers from different regions around the country would have favored a broader vision, making it possible to carry out the analysis based on the type of treatment center and the multidisciplinary approach. Variables such as usual physical activity, age at the initiation of prophylaxis and the intake of analgesics are relevant clinical variables that have not been collected in this study.

### 4.2. Relevance to Clinical Practice

Therapeutic exercise is relevant in gaining strength and improving pain [42]. Physical exercise, in addition to improving muscle strength and coordination, triggers central and peripheral mechanisms that reduce pain [43]. Physiotherapy treatment combined with individualized prophylaxis allows for a wide range of therapeutic options in patients with hemophilia. Low- [44] and high-resistance [45] elastic-band-based training has been shown to be effective in increasing the maximum isometric strength in the lower limbs. A recent study has shown how blood flow restriction training appears to be safe and feasible and does not cause acute or delayed pain in patients with knee arthropathy [46].

## 5. Conclusions

Patients with hemophilia have less quadriceps muscle strength and a lower knee pressure pain threshold than their healthy peers. However, there are no differences in quadriceps muscle activation between patients with hemophilic arthropathy and healthy subjects. Quadriceps muscle fatigue is greater in patients with hemophilic knee arthropathy. Muscle strength training in patients with hemophilia should focus on vastus medialis and lateralis activation.

## Figures and Tables

**Table 1:** Descriptive characteristics of the subjects in the hemophilia and control groups.

Variables	Hemophilia Group (*n* = 23)	Control Group (*n* = 24)	*p*-Value
Age (years)	37.30 (7.04) ^1^	41.50 (8.15) ^1^	0.06 ^2^
Weight (Kg)	80.98 (10.85) ^1^	76.62 (8.12) ^1^	0.12 ^2^
Height (m)	1.73 (0.09) ^3^	1.74 (0.09) ^3^	0.28 ^4^
Body mass index (kg/m^2^)	25.75 (6.3013) ^3^	25.78 (4.43) ^3^	0.44 ^4^
Knee joint damage (0–40)	20.43 (5.19) ^1^	-	-
Lower limb joint damage (0–84)	41.91 (9.32) ^1^	-	-
Knee range of motion, dominant joint (degrees)	124.00 (8.00) ^3^	138.00 (3.00) ^3^	0.00 ^4^
Knee range of motion, non-dominant joint (degrees)	128.00 (6.00) ^3^	138.50 (2.00) ^3^	0.00 ^4^
	*n* (%)		
Type of hemophilia (A/B)	17/6 (73.9/26.1)	-	-
Inhibitor (yes/no)	7/16 (30.4/69.6)	-	-
Treatment (on-demand/prophylaxis)	7/16 (30.4/69.6)	-	-
Type of treatment (SHL/EHL/BMA)	7/9/7 (14.9/19.1/14.9)		

SHL: short half-life; EHL: extended half-life; BMA: bispecific monoclonal antibody. ^1^ Mean (standard deviation) was applied; ^2^ Student’s *t*-test for independent samples was performed; ^3^ median (interquartile range) was used; ^4^ Mann–Whitney U test was utilized.

**Table 2:** Means (standard deviations) and normality and homogeneity analysis of variables between groups.

Variables	Hemophilia Group		Control Group		*p*-Value
	Dominant Joint	Non-Dominant Joint	Dominant Joint	Non-Dominant Joint	Dominant Joint/Non-Dominant Joint
Quadriceps strength (N/cm^2^)	216.70 (56.12) ^1^	224.72 (49.94) ^1^	316.66 (47.97) ^1^	289.31 (42.80) ^1^	**0.00** ^2^/**0.00** ^2^
Pressure pain threshold (kg/cm^2^)	75.93 (33.10) ^1^	64.10 (55.8) ^3^	99.43 (31.96) ^1^	96.95 (53.72) ^3^	**0.01** ^2^/**0.03** ^4^
RMS vastus medialis (microV)	125.12 (63.45) ^3^	121.72 (65.43) ^3^	139.23 (77.41) ^3^	150.89 (91.2) ^3^	0.29 ^4^/0.05 ^4^
RMS vastus lateralis (microV)	154.82 (84.56) ^3^	150.12 (85.66) ^3^	172.90 (71.31) ^3^	153.67 (104.13) ^3^	0.34 ^4^/0.55 ^4^
RMS rectus femoris (microV)	201.52 (161.17) ^3^	198.42 (162.19) ^3^	198.96 (164.19) ^3^	173.88 (82.18) ^3^	0.18 ^4^/0.91 ^4^
VMF vastus medialis (microV)	58.98 (10.31) ^1^	57.08 (11,62) ^1^	57.74 (7.50) ^1^	71.69 (9.59) ^1^	0.64 ^2^/**0.00** ^2^
VMF vastus lateralis (microV)	62.88 (21.97) ^3^	63.13 (17.95) ^1^	61.66 (16.52) ^3^	73.98 (9.81) ^1^	0.79 ^4^/**0.02** ^2^
VMF rectus femoris (microV)	67.79 (11.84) ^1^	65.65 (11.92) ^1^	75.48 (11.12) ^1^	81.28 (14.74) ^1^	**0.02** ^2^/**0.00** ^2^

RMS: root mean square; VMF: variation of median frequency; ^1^ Mean (standard deviation) was applied; ^2^ Student’s *t*-test for independent samples was performed; ^3^ median (interquartile range) was used; ^4^ Mann–Whitney U test was utilized. A *p* < 0.05 with a 95% confidence interval (bold) was considered statistically significant.

**Table 3:** Differences between groups and effect size.

Variables	Joint	MD	CI95%	ES
Quadriceps strength	Dominant joint	100.52	64.69; 129.2	1.38
	Non-dominant joint	65.57	29.95; 93.55	1.14
Pressure pain threshold	Dominant joint	23.02	3.3; 43.54	0.68
	Non-dominant joint	23.45	3.09; 45.25	0.56
RMS vastus medialis	Dominant joint	16.92	−14.47; 45.88	0.37
	Non-dominant joint	31.17	−1.02; 67.62	0.63
RMS vastus lateralis	Dominant joint	16.59	−17.03; 62.68	0.40
	Non-dominant joint	11.98	−32.18; 49.16	0.24
RMS rectus femoris	Dominant joint	46.33	−24.67; 105.5	0.49
	Non-dominant joint	−2.46	−63.86; 73.91	0.07
VMF vastus medialis	Dominant joint	−0.69	−7.23; 5.5	−0.14
	Non-dominant joint	14.46	8.72; 21.51	1.18
VMF vastus lateralis	Dominant joint	0.48	−8.07; 8.07	−0.13
	Non-dominant joint	14.56	4.84; 21.66	0.68
VMF rectus femoris	Dominant joint	7.30	0.76; 14.79	0.64
	Non-dominant joint	15.71	6.48; 24.95	1.01

RMS: root mean square; VMF: variation of median frequency; MD: mean difference; CI95%: 95% confidence interval; ES: effect size.

**Table 4:** Correlation analysis (Rho (significance)) of range of motion, quadriceps strength, pressure pain threshold, root mean square and variation of median frequency.

Group	Variables	ROM	Strength	PPT	RMS-VM	RMS-VL	RMS-RF	VMF-VM	VMF-VL
Hemophilia	Strength	**0.29 (0.04)**							
	PPT	0.17 (0.23)	0.04 (0.78)						
	RMS-VM	0.01 (0.92)	**0.33 (0.02)**	0.09 (0.54)					
	RMS-VL	−0.09 (0.54)	**0.37 (0.01)**	−0.05 (0.69)	**0.71 (0.00)**				
	RMS-RF	−0.10 (0.48)	0.10 (0.49)	0.38 (0.01)	**0.63 (0.00)**	**0.62 (0.00)**			
	VMF-VM	**−0.34 (0.01)**	0.12 (0.40)	0.13 (0.36)	**0.67 (0.00)**	**0.54 (0.00)**	**0.61 (0.00)**		
	VMF-VL	−0.20 (0.17)	0.21 (0.15)	0.06 (0.65)	**0.67 (0.00)**	0.28 (0.05)	**0.53 (0.00)**	**0.77 (0.00)**	
	VMF-RF	−0.17 (0.24)	**0.42 (0.00)**	−0.05 (0.73)	**0.57 (0.00)**	**0.37 (0.01)**	0.24 (0.10)	**0.73 (0.00)**	**0.74 (0.00)**
Controls	Strength	0.02 (0.89)							
	PPT	−0.01 (0.93)	−0.06 (0.67)						
	RMS-VM	−0.09 (0.54)	−0.05 (0.72)	−0.09 (0.53)					
	RMS-VL	−0.17 (0.23)	0.08 (0.58)	−0.08 (0.55)	**0.67 (0.00)**				
	RMS-RF	0.07 (0.61)	−0.04 (0.74)	−0.08 (0.57)	**0.41 (0.01)**	**0.49 (0.00)**			
	VMF-VM	0.13 (0.34)	**−0.29 (0.04)**	−0.09 (0.52)	0.06 (0.64)	−0.12 (0.41)	−0.19 (0.19)		
	VMF-VL	0.06 (0.65)	−0.20 (0.16)	0.03 (0.80)	−0.12 (0.41)	−0.12 (0.40)	**−0.57 (0.00)**	**0.58 (0.00)**	
	VMF-RF	0.09 (0.50)	0.15 (0.30)	−0.11 (0.43)	−0.15 (0.29)	−0.19 (0.19)	−0.26 (0.07)	0.14 (0.34)	0.18 (0.21)

ROM: range of motion; PPT: pressure pain threshold: VM: vastus medialis; VL: vastus lateralis; RF: rectus femoris; RMS: root mean square; VMF: variation of median frequency; A *p* < 0.05 (bold) was considered statistically significant.

## Data Availability

The data that support the findings of this study are available on request from the corresponding authors. The data are not publicly available due to privacy and ethical restrictions.
